# The influence of electromagnetic radiation on the measurement behaviour of the triggerfish® contact lens sensor

**DOI:** 10.1186/s12886-018-1013-x

**Published:** 2018-12-27

**Authors:** Dieter Franz Rabensteiner, Jasmin Rabensteiner, Christoph Faschinger

**Affiliations:** 1 Department of Ophthalmology, Medical University of Graz Auenbruggerplatz 4, 8036 Graz, Austria; 2 Clinical Institute of Medical and Chemical Laboratory Diagnostics, Medical University of Graz, Auenbruggerplatz 15, 8036 Graz, Austria

**Keywords:** Glaucoma, Intraocular pressure, Contact lens, Experimental - laboratory

## Abstract

**Background:**

To assess a possible signal drift, noise and influences of electromagnetic radiation on the measurement behaviour of the Triggerfish® contact lens sensor, which might be mistaken as IOP fluctuations.

**Methods:**

Contact lens sensors (Triggerfish®, SENSIMED AG, Lausanne, Switzerland) were fixed in a water bath. To reduce any external electromagnetic impulses, all plugs were removed from the sockets, no lights were switched on and no electronic devices, except a temperature logger were left in the test room. For 24 h signal drift, noise and the influences of a cordless telephone (Ascom d43 DECT Handset, EU DECT 1880–1900 MHz, Ascom Wireless, Baar, Switzerland), a smartphone (Sony Xperia Go ST27i, Sony Corporation, Tokyo, Japan) and a computer (Hewlett-Packard ProBook 650 15,6″ - D9S33AV, Hewlett-Packard Inc., Palo Alto, USA) on the measuring profile were analysed.

**Results:**

Twenty-four-hour measurements without provoked external electromagnetic impulses yielded a profile without any signal drift and 8.2 mV eq noise. During the activation of the cordless telephone a maximum measurement variation of 3.2 mV eq. (4.1–7.3), smartphone 1.8 mV eq. (4.7–6.5) and computer 1.4 mV eq. (6.3–7.7) were observed.

**Conclusions:**

During 24-h measurements there was no signal drift and a very low noise. Patients concerned about electronic devices possibly interfering with the measurements of the contact lens sensor, can be informed, that the use of their cordless telephone, smartphone or computer does not cause any problems. The amount of the signal noise might help to define actual IOP fluctuations. Temperature fluctuations might influence the measuring profile.

## Background

The main risk factor for glaucoma diseases is an increased intraocular pressure (IOP) exceeding an individual threshold. IOP is usually measured during office times one or more times while sitting, rarely in supine position and/or at night. Several measurements over the 24-h period would make sense since the IOP is not a constant, but an individually variable measured value with a minimum and a maximum [1]. These changes in the daytime are called fluctuations, and their causes may be manifold. Short-term fluctuations are distinguished from long-term fluctuations. Fluctuations in glaucoma patients are per se higher than in healthy subjects, especially in intermittent angle block disease. Whether they actually represent a separate risk factor regarding the development and/or progression of glaucoma is discussed controversially [2–4].

Therefore, it is of great interest to know the course of the IOP during the whole day and night [5]. Nevertheless, a high number of measurements over the 24-h period is difficult to carry out, and night measurements are only feasible in specially equipped sleep laboratories. In 2009 the contact lens sensor Triggerfish® (SENSIMED AG, Lausanne, Switzerland) was launched for “continuous measurement of the eye pressure” [6]. In the meantime, there are numerous positive [7–10] but also critical publications [11–16] on the results of this measurement method. The contact lens sensor provides a curve of numerous measuring points, which are obtained over 24 h. It is still not defined, what amount of change in the 24-h profile is an IOP fluctuation or signal noise.

During the 24-h measurements, all usual activities of the day should be carried out and recorded in a written diary, with the exception of swimming, bathing and showering, as incoming water might damage the recorder and wearing glasses with metal frames.

The aim of this study was to assess, whether there is a significant level of signal drift or noise during the 24-h measuring period preventing the identification of IOP fluctuations. Furthermore, since some electronic devices, such as a telephone or a computer are used by the patients during the measurement period and also parts of the Triggerfish® equipment (e.g. the antenna and the recorder) are electronic devices themselves, we assessed, whether the active use of “daily-use” electronic devices by the patient during the measurement period influences the measuring profile and therefore might be mistaken as IOP fluctuation.

## Methods

Two new Triggerfish® contact lens sensors were fixed in their original holders in a water bath (simulating the moist, fluid environment of the tear film). The antenna for wireless recording and output of data was positioned at approximately the same distance as from the orbital bones to the eyeball (around 1.5 cm). After the start, for 24 h measurements were taken every 5 min for 30 s, as programmed by the company SENSIMED AG, “pseudo-continuously”.

### Detection of possible signal drift or noise

In order to eliminate any disturbing electromagnetic impulses, all plugs were removed from the sockets in the test room, no lights (especially no neon lights) were switched on, no telephones (fixed line, cordless, smartphone) and no computer were left in the room. In order to investigate a possible influence of temperature changes in the room a temperature logger was positioned directly next to the measuring setup.

### Influence of electronic devices

**Fig. 1:**
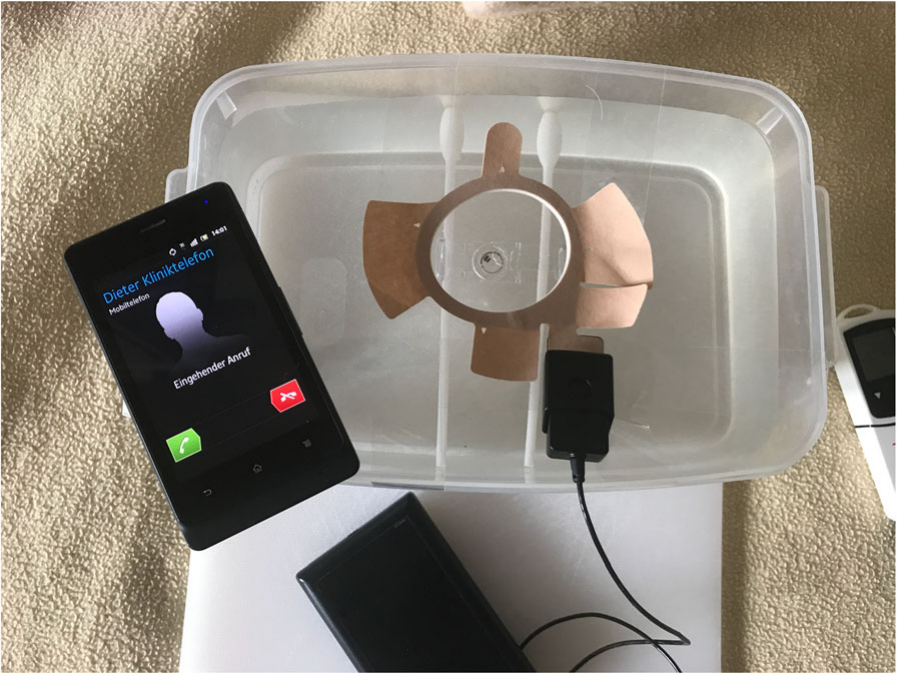
Test setup: Triggerfish® contact lens sensor in the original container floats superficially in the water bath 1.5 cm beneath the circular antenna (beige colour) for wireless recording of the measured data and emission of measuring pulses. The antenna is connected to the data recorder with a wire. In about 10 cm distance to the antenna an active smartphone is positioned, receiving a call, lasting for the 47 min

Like in the first setup, all plugs were removed from the sockets and no lights were switched on. After a “consolidation time” of one hour, a cordless telephone (Ascom d43 Digital Enhanced Cordless Telecommunications (DECT) Handset, EU DECT 1880–1900 MHz, Ascom Wireless, Baar, Switzerland) was positioned 10 cm next to the contact lens sensor and switched on for one hour (in conversation mode with another telephone). The same was carried out after a two-hour break with a smartphone (Sony Xperia Go ST27i, Sony Corporation, Tokyo, Japan) for 47 min in conversation mode, which can be seen in Fig. 1. After a 30-min break, a laptop (Hewlett-Packard ProBook 650 15,6″ - D9S33AV, Hewlett-Packard Inc., Palo Alto, USA) was moved about 20–30 cm to the contact lens sensor and used for 45 min for word processing and various email as well as internet activities via a wireless internet connection (2.4Ghz).

## Results

Both measurements ran without technical problems, the profiles were well retrieved from the recorder.

**Fig. 2:**
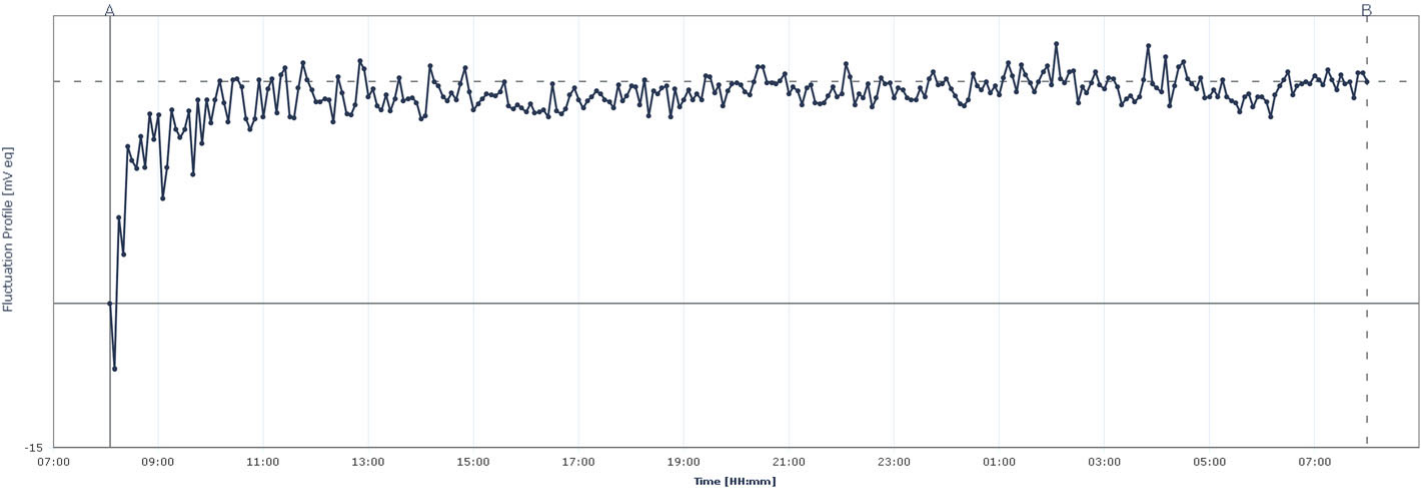
24-h measurement profile of the contact lens sensor in the water bath without external electromagnetic interference to assess signal drift and noise. The profile is flat, the fluctuations between the measurements are between the minimum of 18.8 mV eq at 10:00 o’clock and the maximum of 27.0 mV eq at 02:05 on the next day (8.2 mV eq difference, noise). In the first two hours, an “adaptation” took place, in which the curve increases for an unknown cause. X-axis time, Y-axis from − 15 to 30 mV eq.

**Fig. 3:**
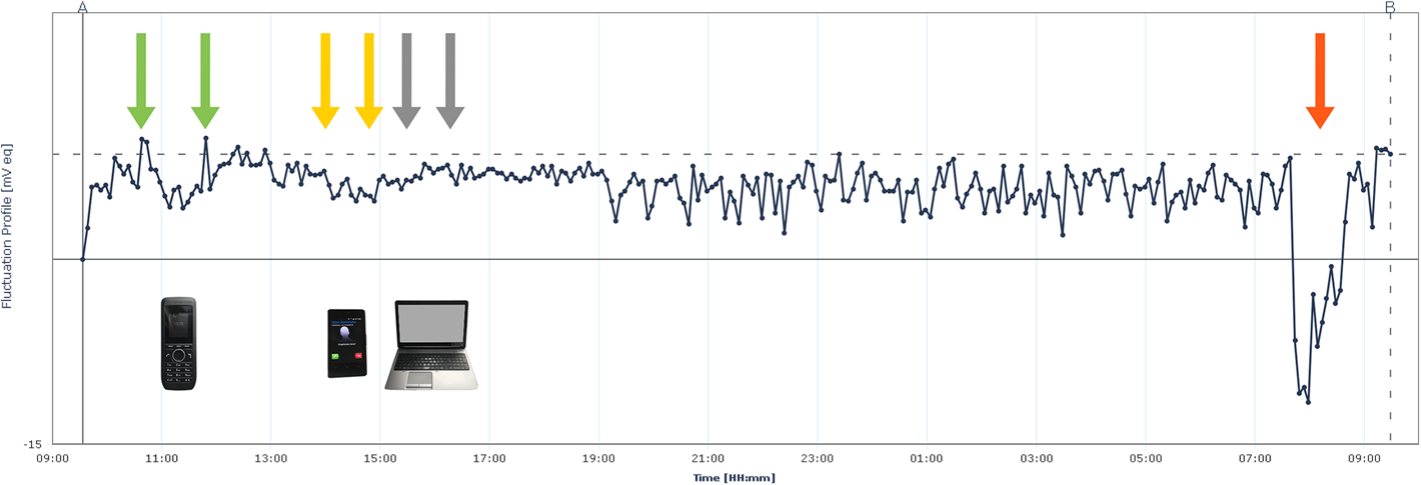
Measuring profile of the contact lens sensor in the water bath in order to investigate possible electromagnetic influences from activated devices (cordless telephone 58 min active, green arrows, smartphone 47 min active, yellow arrows, laptop with wireless internet connection 45 min active, grey arrows). The profile is completely flat and not influenced by these devices. The fluctuations between the measurements lie between the minimum of 1.9 mV eq at 3:28 h and the maximum of 9.8 mV eq at 11:48 h (7.9 mV eq difference, noise). Only by a change of the room temperature (a decrease of 5° Celsius), there is a drop in the profile (red arrow, difference 19.2 mV eq). X-axis time, Y-axis from − 15 to 20 mV eq.

1. After the start (08:05 with 0 mV eq = microVolt equivalent), the sensor took about 2 h to reach the steady-state level (average ~ 22 mV eq), which was maintained over the next 22 h. The 22-h measurements yielded a flat profile without drift (direction up or down). The fluctuations during the measurements were between 1 and 2.5 mV eq, between the individual measurements from the minimum 18.8 mV eq up to the maximum of 27.0 mV eq. (8.2 mV eq difference, noise) (Fig. 2.). The temperature fluctuations in the room were around one degree of Celsius.

2. The measurements during the tests (09:33–07:38 on the next day) generated a flat profile within 21.5 h (Fig. 3). After the start (09:33), the sensor needed 30 min to reach the steady-state level (on average 7.5 mV eq), which was maintained during the next 21.5 h. The fluctuations during the measurements were between 1.0 and 2.5 mV eq. Activation of the cordless telephone took place between 10:48 and 11:46 (active for 58 min), with a maximum variation of the measured values of 3.2 mV eq. (4.1–7.3, within 12 measurements). The activation of a smartphone took place between 14:05 and 14:52 (active for 47 min), with a maximum variation of the measured values of 1.8 mV eq. (4.7–6.5, within 8 measurements). The laptop with a wireless internet connection was switched on at 15:30 and switched off at 16:15 (45 min of use). The maximum variation of the measured values was 1.4 mV eq. (6.3–7.7, within 10 measurements). Until that, the temperature fluctuations in the room were around one degree of Celsius. Due to a provoked decrease of the room temperature by 5° Celsius at the end of the measuring period, the profile changed significantly (19.2 mV eq).

## Discussion

The IOP as the main risk factor for the development and progression of glaucoma is not a constant value over 24 h and varies individually [4]. These fluctuations are termed short-term and long-term fluctuations. They may be circadian, but also influenced by the position of the head (upright, flat or downward), by activities (headstands in yoga, playing of wind instruments, etc.). Therefore, multiple measurements of the IOP, preferably at regular short intervals or continuously - similar to the 24-h electrocardiogram or 24-h blood pressure measurements - are of interest in order to be able to detect these fluctuations. Repeated eye pressure measurements with the Goldmann applanation tonometer or air-pulsed tonometers would be possible, but practically speaking they are too burdensome for the eye and the patient. Particularly at night, they are difficult to be carried out.

For this purpose, the Swiss company SENSIMED AG has developed a CE-certified and FDA-approved contact lens sensor [6], which carries out 24-h pseudo-continuous measurements (every 5 min for 30 s) and as a result provides a 24-h measuring profile. The contact lens sensor, the associated antenna and the recording device are adjusted on an outpatient basis and the measurement is prepared and started by the bundled software. The data transfer is carried out wirelessly via a self-adhesive antenna that is positioned around the orbit. During the 24-h measuring period, the patients should perform all of their usual activities. These are recorded in a written diary in order to be able to relate any changes in the 24-h measurement profile. For example, the beginning and the end of times in supine position should be noted in order to be able to correlate the associated physiological pressure increase as a result of the change in position with an increase in the profile [17, 18]. The same applies to the application of antiglaucomatous drugs and their associated (expected) decrease in the profile.

Nowadays, more and more electronic devices are used in everyday life. One of the largest sources of electromagnetic radiation being the antennas of cordless telephones, smartphones and computers. These could potentially influence the wireless data transfer of the contact lens sensor to the antenna by emitting high-frequency pulsed electromagnetic radiation and therefore interfere with the identification of IOP fluctuations.

We investigated, whether there is a significant level of drift or noise during the 24-h measuring period, since noise limits sensor sensitivity. The analysis of our data did not show a signal drift within the 22-h measurement period and we could only detect a very low noise of 8.2 mV eq. The assessment of the noise of the contact lens sensor could result in a uniform definition of significant short-term fluctuations (“peaks”) as an actual change, of e.g. at least two times the extent of the noise (> 16 mV eq).

Within the second experiment, we brought a cordless telephone with DECT technology (active for 58 min), a smartphone (47 min conversation) and a laptop with active wireless internet connection (used for 45 min) into the usual conversation or working distance to the contact lens sensor. We did not detect any variations in the measurement profile, which were larger than the noise from our first experiment. The fluctuations between the lowest and the highest value were 7.9 mV eq. According to the manufacturer of the contact lens sensor Triggerfish®, no spectacles with metal frames may be worn during the 24-h measurement, since these could interfere with the data transmission. In addition, visits to the sauna (excessive sweat, moisture), bathing and showers (water could detach the antenna from the skin or destroy the data recorder) should be avoided. Nevertheless, patients concerned about electronic devices or “electrosmog” possibly interfering with the measurements of the contact lens sensor, can be informed, that the use of their cordless telephone, smartphone or computer does not cause any problems.

At the end of the second experiment, we could once again prove the known disturbance of the measurement profile by changes of the room temperature [19]. The in vitro experiments were performed in a well-controlled environment and have shown there is no influence from electronic devices on the measuring profile of the contact lens sensor. Whether the in vivo use of electronic devices can induce alterations of the measuring profile by the generation of heat to organ tissue has to be investigated in another testing environment. Studies to which extent the changes of ambient air temperature can interfere with the measurement profile (e.g. especially in winter when leaving a warm living room outside into the cold) are warranted.

## Conclusions

The contact lens sensor Triggerfish® shows no signal drift and a very low noise. Patients concerned about electronic devices possibly interfering with the measurements of the contact lens sensor, can be informed, that the use of their cordless telephone, smartphone or computer does not cause any problems. Temperature fluctuations might influence the measuring profile.

## Abbreviations

DECT: Digital Enhanced Cordless Telecommunications
IOP: Intraocular pressure
mV eq: microVolt equivalent

## References

[1] Göbel K, Rüfer F, Erb C (2011). Physiology of aqueous humor formation, diurnal fluctuation of intraocular pressure and its significance for glaucoma. Klin Monatsbl Augenheilkd.

[2] Leidl MC, Choi CJ, Syed ZA, Melki SA (2014). Intraocular pressure fluctuation and glaucoma progression: what do we know?. Br J Ophthalmol.

[3] Caprioli J, Coleman AL. Intraocular pressure fluctuation a risk factor for visual field progression at low intraocular pressures in the advanced glaucoma intervention study. Ophthalmology 2008;115:1123–3. 10.1016/j.ophtha.2007.10.031 18082889

[4] Quaranta L, Riva I, Oddone F (2016). 24-hour IOP fluctuation: myth or reality?. J Mod Ophthalmol.

[5] Sit AJ (2009). Continuous monitoring of intraocular pressure: rationale and progress toward a clinical device. J Glaucoma.

[6] Leonardi M, Pitchon EM, Bertsch A, Renaud P, Mermoud A (2009). Wireless contact lens sensor for intraocular pressure monitoring: assessment on enucleated pig eyes. Acta Ophthalmol.

[7] Mansouri K, Shaarawy T (2011). Continuous intraocular pressure monitoring with a wireless ocular telemetry sensor: initial clinical experience in patients with open angle glaucoma. Br J Ophthalmol.

[8] Mansouri K, Medeiros FA, Tafreshi A, Weinreb RN (2012). Continuous 24-hour monitoring of intraocular pressure patterns with a contact lens sensor: safety, tolerability, and reproducibility in patients with glaucoma. Arch Ophthalmol.

[9] Mansouri K, Weinreb RN, Liu JHK (2015). Efficacy of a contact lens sensor for monitoring 24-h intraocular pressure related patterns. Lin H, editor PLoS ONE.

[10] Dunbar GE, Shen BY, Aref AA (2017). The Sensimed triggerfish contact lens sensor: efficacy, safety, and patient perspectives. Clin Ophthalmol.

[11] Faschinger C, Mossböck G, Validity KS (2012). Reproducibility of sensor contact Lens profiles in comparison to Applanation tonometry in healthy eyes. Klin Monatsbl Augenheilkd.

[12] Faschinger C, Mossböck G (2013). Validity of the results of a contact lens sensor?. JAMA Ophthalmol.

[13] Holló G, Kóthy P, Vargha P (2014). Evaluation of continuous 24-hour intraocular pressure monitoring for assessment of prostaglandin-induced pressure reduction in glaucoma. J Glaucoma.

[14] Sunaric-Megevand G, Leuenberger P, Preußner P-R (2014). Assessment of the triggerfish ™contact lens sensor for measurement of intraocular pressure variations. Acta Ophthalmol.

[15] Faschinger C (2016). Contact Lens sensor triggerfish – what do we know?. European Ophthalmic Review.

[16] Vitish-Sharma P, Acheson AG, Stead R, Sharp J, Abbas A, Hovan M (2017). Can the SENSIMED triggerfish(®) lens data be used as an accurate measure of intraocular pressure?. Acta Ophthalmol.

[17] Anderson DR, Grant WM (1973). The influence of position on intraocular pressure. Investig Ophthalmol.

[18] Prata TS, De Moraes CGV, Kanadani FN, Ritch R, Paranhos A (2010). Posture-induced intraocular pressure changes: considerations regarding body position in glaucoma patients. Surv Ophthalmol.

[19] Faschinger CW, Rabensteiner DF, Mossböck G (2014). How do temperature variations influence the signal in the triggerfish contact lens sensor?. Spektrum Augenheilkd.

